# Predicting acute termination and non-termination during ablation of human atrial fibrillation using quantitative indices

**DOI:** 10.3389/fphys.2022.939350

**Published:** 2022-11-22

**Authors:** Cole Kappel, Michael Reiss, Miguel Rodrigo, Prasanth Ganesan, Sanjiv M. Narayan, Wouter-Jan Rappel

**Affiliations:** ^1^ Department of Physics, UC Irvine, Irvine, CA, United States; ^2^ Department of Physics, UC San Diego, La Jolla, CA, United States; ^3^ CoMMLab, Electronic Engineering Department, Universitat de Valencia, Valencia, Spain; ^4^ Stanford Cardiovascular Institute, Stanford University School of Medicine, Stanford, CA, United States

**Keywords:** atrial fibrillation, ablation, termination, computational analysis, electrograms

## Abstract

**Background:** Termination of atrial fibrillation (AF), the most common arrhythmia in the United States, during catheter ablation is an attractive procedural endpoint, which has been associated with improved long-term outcome in some studies. It is not clear, however, whether it is possible to predict termination using clinical data. We developed and applied three quantitative indices in global multielectrode recordings of AF prior to ablation: average dominant frequency (ADF), spectral power index (SPI), and electrogram quality index (EQI).

**Methods:** In N = 42 persistent AF patients (65 ± 9 years, 14% female) we collected unipolar electrograms from 64-pole baskets (Abbott, CA). We studied N = 17 patients in whom AF terminated during ablation (“Term”) and N = 25 in whom it did not (“Non-term”). For each index, we determined its ability to predict ablation by computing receiver operating characteristic (ROC) and calculated the area under the curve (AUC).

**Results:** The ADF did not differ for Term and Non-term patients at 5.28 ± 0.82 Hz and 5.51 ± 0.81 Hz, respectively (*p* = 0.34). Conversely, the SPI for these two groups was. 0.85 (0.80–0.92) and 0.97 (0.93–0.98) and the EQI was 0.61 (0.58–0.64) and 0.56 (0.55–0.59) (*p* < 0.0001). The AUC for predicting AF termination for the SPI was 0.85 ([0.68, 0.95] 95% CI), and for the EQI, 0.86 ([0.72, 0.95] 95% CI).

**Conclusion:** Both the EQI and the SPI may provide a useful clinical tool to predict procedural ablation outcome in persistent AF patients. Future studies are required to identify which physiological features of AF are revealed by these indices and hence linked to AF termination or non-termination.

## Introduction

Atrial fibrillation (AF) is the most common cardiac arrhythmia in the world and currently affects more than five million people in the United States alone (Chugh et al., 2014; Calkins et al., 2018). AF is associated with increased risk for stroke and morbidity. There is increasing evidence that early intervention for AF, either by drug therapy or ablation, can reduce long-term adverse outcomes (Kirchhof et al., 2020), yet both forms of therapy are suboptimal. Pulmonary vein isolation (PVI) to electrically isolate the pulmonary veins is the cornerstone of ablation treatment (Haissaguerre et al., 1998), but is only modestly effective with a 1-year success rate in persistent AF patients of 50–60% (Calkins et al., 2017). Several additional ablation procedures have been proposed yet with varying outcomes, such as targeting complex fractionated electrograms (Nademanee et al., 2004; Knecht et al., 2008), rotational and focal sources (Narayan et al., 2012; Ramirez et al., 2017; Baykaner et al., 2018), posterior wall isolation (Lee et al., 2019; Pothineni et al., 2021; Chieng et al., 2022) and others. It would be useful to track AF ablation intraprocedurally, to determine if the current strategy is effective in this patient or whether additional lesions are warranted.

Currently, there is no quantitative metric that can reliably predict whether ablation will result in AF termination or not. Such a metric may be procedurally useful. In addition, some studies have shown that acute termination during ablation is associated with improved long-term outcome (O'Neill et al., 2009; Scherr et al., 2015; Heist et al., 2012; Zhou et al., 2013; Park et al., 2012; Schreiber et al., 2015; Rostock et al., 2011). Furthermore, a reliable metric may provide insights into the spatio-temporal activation patterns that underlie AF.

In this study, we developed and compared the ability of three different quantitative indices to predict whether a uniform ablation strategy resulted in AF termination to either sinus rhythm (SR) or atrial tachycardia (AT), or failed to terminate AF. Our indices were computed using unipolar electrograms from 64-pole baskets, measured over a prolonged time (60 s). The indices consisted of the average dominant frequency (ADF), the DF averaged over all electrograms, the spectral power index (SPI), a measure of the power contained in the power spectrum close to the dominant frequency relative to the total power in the spectrum, and a novel electrogram quality index (EQI), a measure of the relative amplitude of the most prominent peak of the time derivative of electrograms in repeated intervals. These indices were tested in N = 42 patients, divided into N = 17 patients that did (“Term”) and N = 25 patients that did not (“Non-term”) terminate during the procedure.

## Methods

We studied 42 patients with persistent AF (defined as patients in whom AF lasted longer than 7 days) referred for ablation at Stanford University Hospital, Palo Alto, CA for standard indications. Of these patients, N = 17 terminated acutely during the ablation procedure, while N = 25 did not. All patients had failed at least one anti-arrhythmic medication, were >18 years and none had contra-indications to ablation. All patients provided written informed consent and this study was approved by our Institutional IRB.

### Data acquisition

No electrical cardioversion was applied at the beginning of the procedure and patients who presented in sinus rhythm were paced into AF. AF was mapped using 64 pole contact baskets (FIRMap, Abbott) for 60 s. The baskets were positioned in LA for AF mapping, based upon 3-dimensional electroanatomic imaging (NavX, St Jude Medical, Sylmar, CA; or Carto, Biosense-Webster, Diamond Bar, CA). This catheter consists of eight splines, each with eight electrodes, totaling 64 electrodes, which improves over older designs and covers >70% of the LA (Honarbakhsh et al., 2017). Within a spline, electrodes are separated by 4–6 mm, and spacing between splines is mostly within 20% of that range (Honarbakhsh et al., 2017). Ablation was guided prospectively at regions of interest identified by a commercial system (RhythmView™, Abbott, Inc.).

Endocardial ablation was used using 3.5 mm irrigated-tip catheters (SmartTouch^®^, Biosense Webster; FlexAbilityTM, Abbott) targeting 30–35 W at 10–20 g force. The primary goal was to perform pulmonary vein isolation assessed by the endpoint of entrance block. Additional lesions were patient-tailored. Selected sites were ablated to cover 2–3 cm regions, to an endpoint of voltage < 0.5 mV. Ablation at any site was abandoned if the esophagus was heated by > 1.5 C despite reduced power or high power short-duration lesions (50W, 6 s), or that overlay regions of phrenic nerve capture. Electrical cardioversion was applied if AF had not terminated (non-termination group).

### Data export

Unipolar electrograms were recorded at 1 kHz sampling and the QRS complex was removed by computing an average QRS complex and subtracting it from electrograms as detailed before (Alhusseini et al., 2017).

### Data analysis

The Dominant Frequency (DF) was computed for each electrode from the Power Spectral Density (PSD) of the unipolar electrogram using a Welch Periodogram (50% overlapping, 4 s length Hamming window) and a frequency interval between 0 and 20 Hz. The ADF for each patient was then computed as the mean of the DF of all 64 electrodes.

**FIGURE 1:**
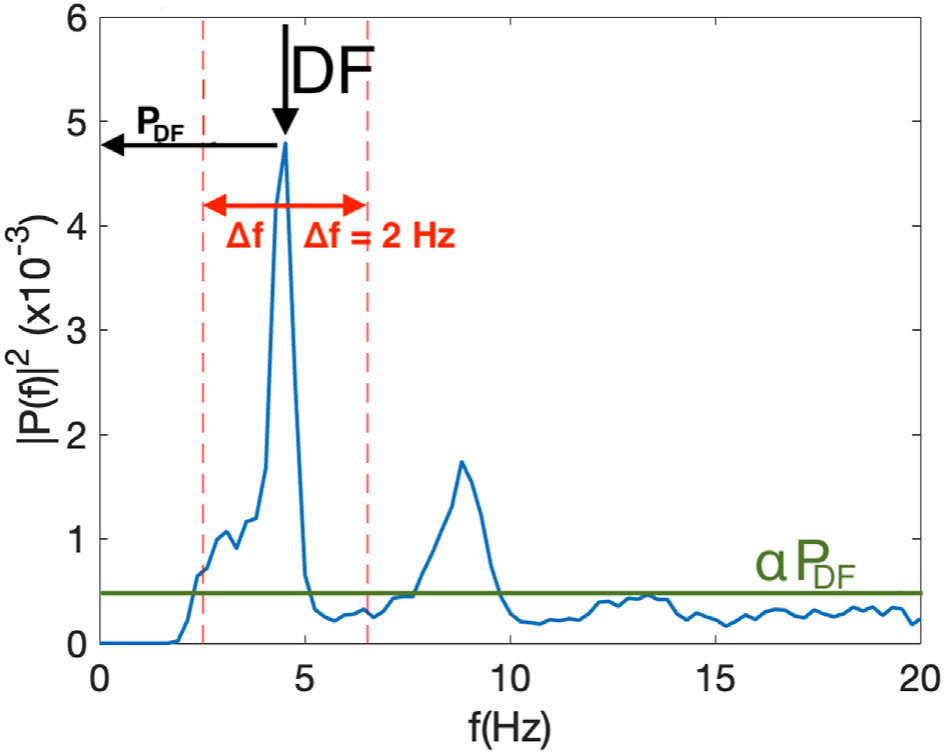
Example of the single sided power spectrum of one of the electrograms used to calculate the DF and the SPI. The threshold, a fraction α of the peak at the DF, αP_DF_, is plotted as a green line using α = 0.1 while the interval around the DF, indicated by the arrow, is plotted as dashed red lines, and shown using Δf = 2 Hz. In this example, SPI = 0.67 and DF = 4.53 Hz.

**FIGURE 2:**
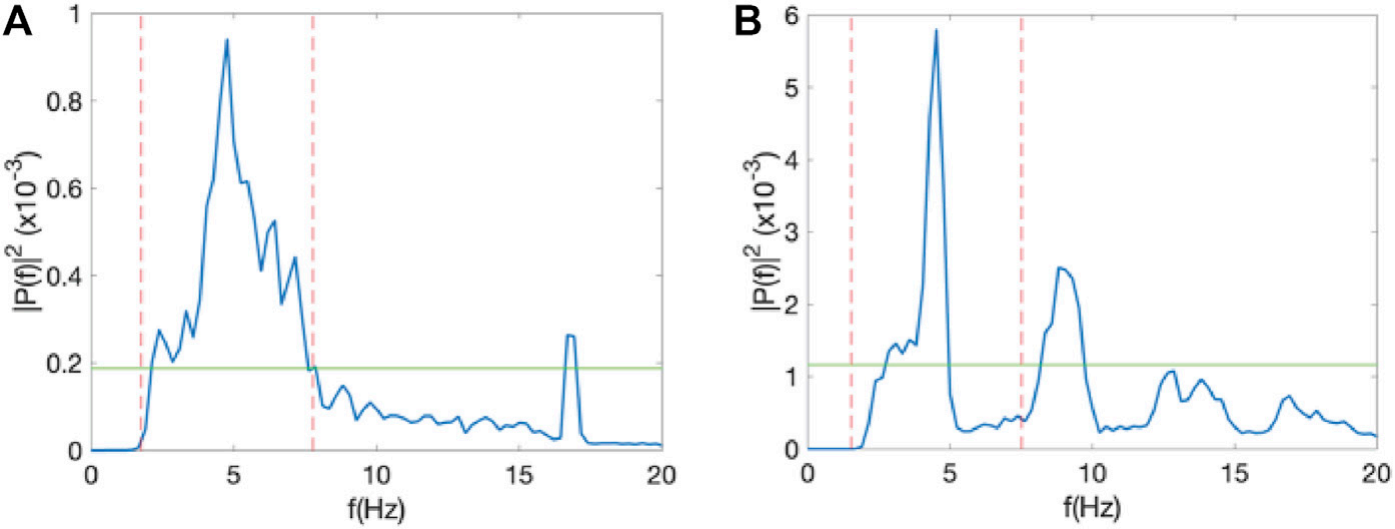
SPI analysis. **(A)** Power spectrum of one of the electrodes of a 55 year-old female in which AF did not terminate during the procedure, corresponding to an SPI value of 0.95, computed using α = 0.2 and Δf = 3 Hz. **(B)** As in A, but for a 51 year-old male in which AF terminated during the procedure with an SPI value of 0.65.

To compute the Spectral Power Index (SPI) of an electrogram (Figure 1), we first determined the spectral power of the DF (P_DF_) and defined a threshold αP_DF_, where α is a value between 0 and 1. This threshold was introduced to remove the noise floor in the signal. Second, we determined all frequencies with a power larger than αP_DF_ and within an interval of width 2Δf symmetrically located around the DF: DF±Δf. The sum of the power for these frequencies was computed as P_int_. The SPI was then defined as the ratio of P_int_ and the sum of the power for all frequencies between 0 and 20 Hz that were above αP_DF_, P_all_: SPI = P_int_/P_all_. This procedure is shown in Figure 1, where we have plotted a sample power spectrum of one of the electrograms. The threshold, here taken to be *α* = 0.1, is shown as a green line while the interval around the DF is indicated as dashed red lines and shown using Δf = 4 Hz. Note that the SPI can take on values between 0 and 1. Specifically, for noisy signals, with a broad peak, we expect that most power is concentrated around the DF. This signal will have a high SPI value. For more regular signals, we expect a narrow peak and more power in harmonics (corresponding to whole number multiples the peak frequency), resulting in a low SPI value. Figure 2A shows a power spectrum of an electrogram with a high SPI (SPI = 0.95). In this case, all power above the threshold, here chosen to be *α* = 0.2, resided within the interval DF±Δf, with Δf = 3 Hz. As a comparison, Figure 2B shows a power spectrum of an electrogram with the low SPI value (SPI = 0.65). In this case, for the same values of the parameters α and Δf, significant amount power is distributed at frequencies outside the interval around DF.

**FIGURE 3:**
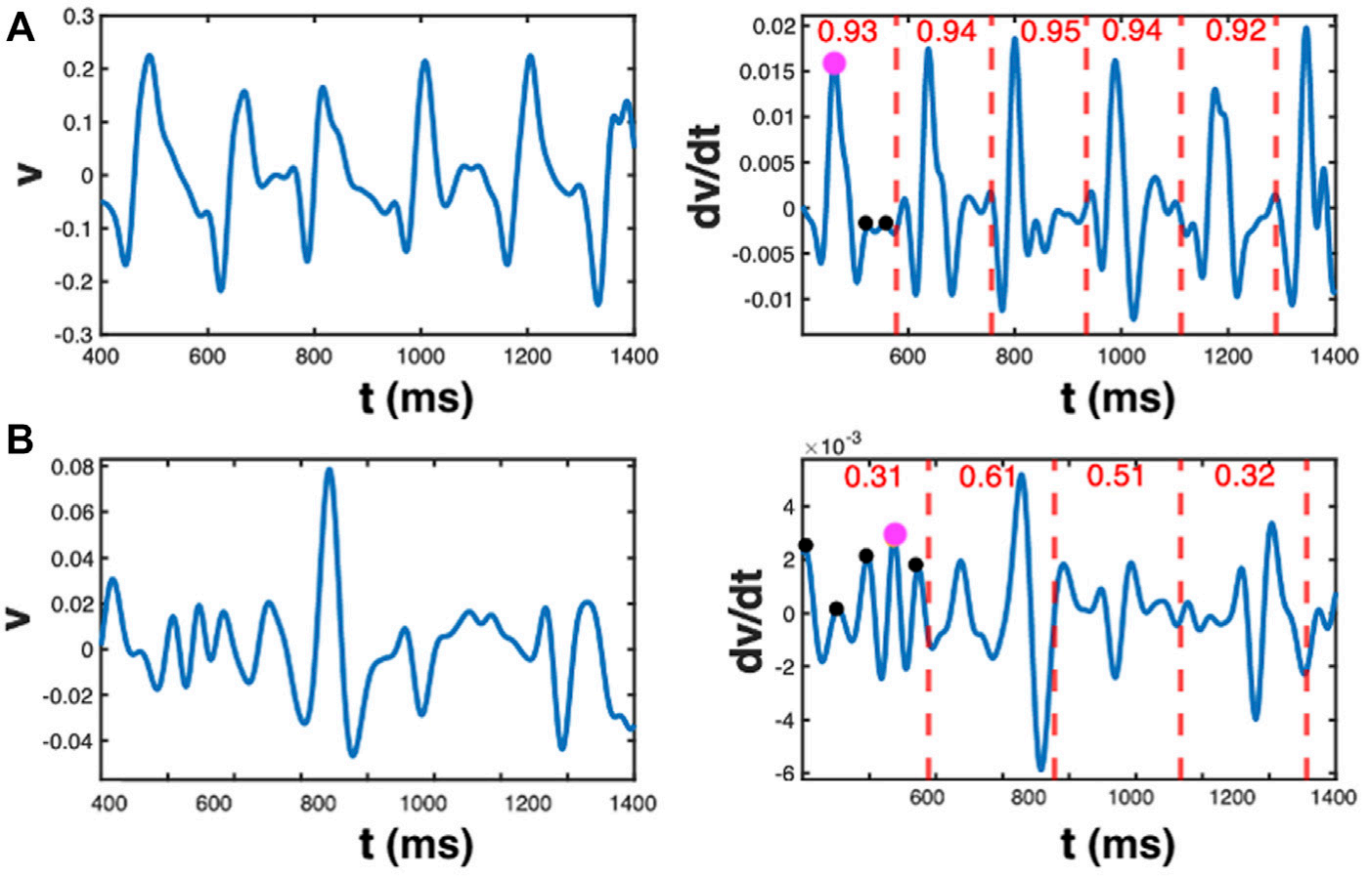
EQI analysis. **(A)** Unipolar trace (left panel) and its time derivative (right panel) for an electrogram with large values of *Q*_*i*_. The value of *Q*_*i*_ is reported in the right panel for each time interval, indicated by the red dashed lines. It quantifies the normalized difference between the maximum value of dv/dt and the sum of all other positive maxima within the interval. These dots are marked for the first interval in magenta and black, respectively. The EQI is computed as the average value of *Q*_*i*_ for each time interval. **(B)** As in A, but now for an electrogram with small values of *Q*_*i*_ (reported in the right panel).

For the Electrogram Quality Index (EQI), we processed each electrogram v(t) with a bandpass filter from 2.5 Hz to 30 Hz as a combination of a low-pass and a high-pass Butterworth filter of fourth order, respectively. Additionally, we applied a fourth order Butterworth notch filter between 55 and 65 Hz to eliminate 60 Hz noise. To calculate the period T of the signal, we used the first peak in the autocorrelation at non-zero time of v(t), smoothed using a zero-phase digital filter. We computed the first derivate with respect to time, $\frac{dv}{dt}$ , and the resulting time trace was divided into N consecutive intervals with width T. This procedure is illustrated in Figure 3 for two sample electrograms and where the intervals are marked by dashed red lines. In these, and other patients, T was around 200 ms, resulting in approximately 300 intervals. We computed for each interval *i* the difference, $Q_{i}$ , between the maximum value of $\frac{dv}{dt}\;,\;\beta ,$ and the sum of all other (n) positive maxima within that interval, γ , normalized by β : $Q_{i}=\left(\beta -\frac{1}{n}\overset{n}{\underset{j=1}{\sum }}\gamma_{j}\right)/\beta$ . In Figure 3, the maximum value β for the first intervals is marked by a magenta dot, while all other positive maxima in the same interval are marked by black dots. For an electrogram with a single peak in its time derivate, the value of $Q_{i}$ will equate to 1. On the other hand, a value of 0 corresponds to a signal with equal valued maxima of $\frac{dv}{dt}$ in the interval. Thus, $Q_{i}$ quantifies the ease with which a large $\frac{dv}{dt}$ can be identified in the interval. Examples of intervals with large values of $Q_{i}$ are shown in Figure 3A: for every interval, the maximum $\frac{dv}{dt}$ is well separated from smaller peaks in $\frac{dv}{dt}$ and clearly distinguishable. As a result, $Q_{i}$ , which quantifies the normalized difference between the value of this peak and the sum of all other positive peaks within each interval, is close to one In contrast, the intervals presented in Figure 3B all have multiple peaks with almost identical amplitudes, resulting in much smaller values of $Q_{i}$ .

The electrogram quality index was then calculated as the average of $Q_{i}$ over all N intervals: $EQI=\sum_{i=1}^{N}Q_{i}/N$ . In summary, the EQI can also take on values between 0 and 1 with large values of EQI corresponding to $\frac{dv}{dt}$ traces with a clear maximum in each interval and small values corresponding to signals that have multiple and almost equally valued peaks in $\frac{dv}{dt}$ for the majority of its time.

### Statistics

Data are reported as mean ± standard deviation for normally distributed data and statistical significance was calculated using a two tailed *t*-test. For data that were not normally distributed, data are reported as median (interquartile 1 - inter quartile 3) and the significance was evaluated with the Wilcoxon rank sum test using the “ranksum” function in MATLAB. A *p*-value < 0.05 was considered statistically significant. Receiver Operating Characteristic Curves (ROC curves) were computed using the built in MATLAB “perfcurve” function and confidence intervals (CIs) of the area under curve (AUC) values were estimated using a non-parametric bootstrap algorithm, using 10,000 iterations.

As a sensitivity analysis, the AUC values were also computed by performing 5,000 iterations in which 5 randomly selected patients were removed from the total cohort. Results form this analysis are reported as median (interquartile 1 - inter quartile 3). Finally, the ROC analysis was repeated by removing outliers in the data set, defined as values that were either 3 standard deviations from the mean (for normal distributions) or 1.5 times the interquartile range (IQR) above the third quartile or below the first quartile (for non-normal distributions).

## Results

### Patient demographics

Table 1 provides the clinical details for the patients in the termination and non-termination cohort. The two groups did not differ significantly in any characteristic.

**TABLE 1:** Patient details.

	*Term (n = 17)*	*Non-term (n = 25)*	*p-value*
Age in years	62.08 ± 10.98	66.92 ± 8.02	0.11
Female	17.6% (3)	12% (3)	0.61
Non-paroxysmal AF	100% (17)	100% (25)	1
Normal LA Size	29.4% (5)	12% (3)	0.16
LVEF %	52.88 ± 12.93	55.04 ± 12.44	0.60
Hypertension	64.7% (11)	60% (15)	0.76
Coronary artery disease	5.88% (1)	24% (6)	0.12
Diabetes mellitus	29.4% (5)	16% (4)	0.30
Transient ischemia attack/stroke	0% (0)	8% (2)	0.50
CHADS2-VASc	1.82 ± 1.33	2.32 ± 1.46	0.27
Previous AF ablation	52.94% (9)	32% (8)	0.18
On Anti Arrhythmic Drug(s)	64.7% (11)	44% (11)	0.19

### Average dominant frequency (ADF)

**FIGURE 4:**
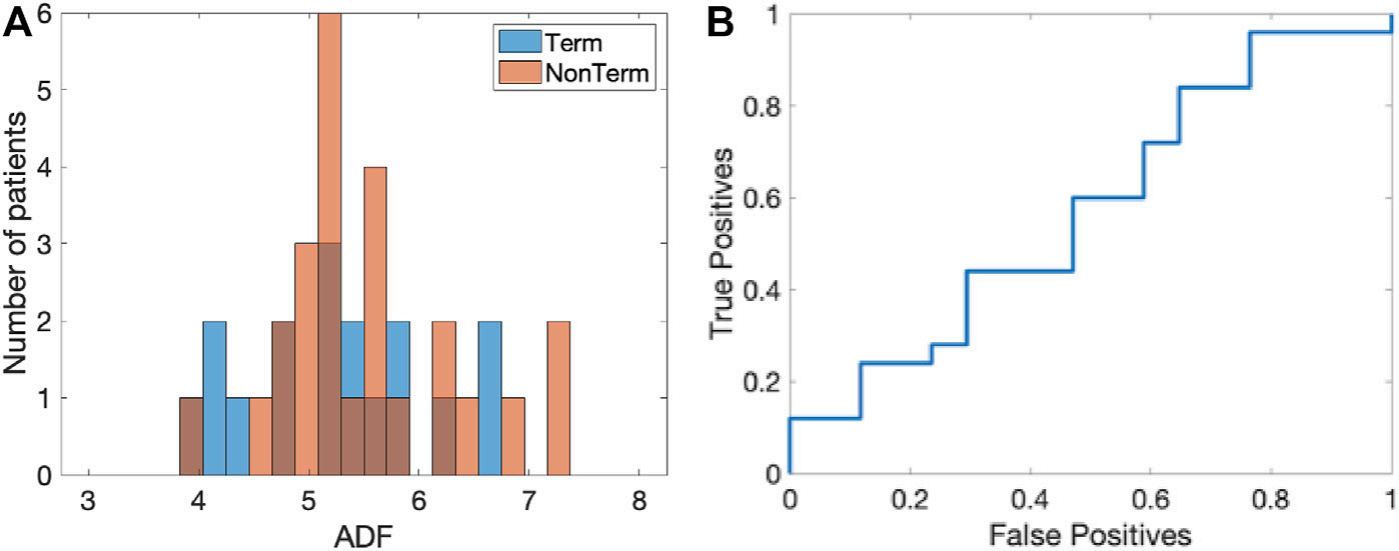
ADF analysis. **(A)** Histogram of the ADF value for the Term (blue) and Non-term groups (red). **(B)** Corresponding ROC curve with an AUC = 0.57 ([0.38, 0.75] 95% CI).

We first determined the ADF using the PSD of the electrograms. The histograms of the ADF values, averaged over all electrograms for each patient, are shown in Figure 4A for both the Term (blue) and Non-term groups (red). For the termination patients, we found that the ADF was 5.28 ± 0.82 Hz while for the non-termination patients this value was 5.51 ± 0.81 Hz (*p* = 0.34). These histograms did not contain any outliers (Methods).

To determine whether this quantity was able to distinguish between termination and non-termination patients, we computed the corresponding receiver operating characteristic (ROC) (Figure 4B). We calculated the area under the curve (AUC) and found that it was 0.57 ([0.38, 0.75] 95% CI). The sensitivity analysis (Methods) resulted in a median AUC that was unaltered, with a small IQR: 0.57 (0.55–0.60).

### Spectral power index (SPI)

We next determined whether the shape of the peak in the power spectrum was significantly different between the termination and non-termination patients. For this, we computed the SPI, which ranges between 0 and one and which represents a measure of the power around the DF relative to the total power (Methods).

**FIGURE 5:**
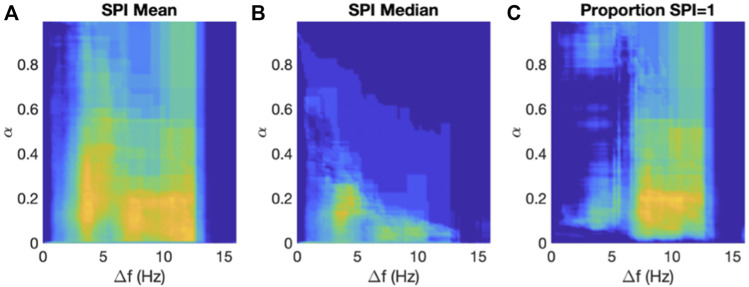
Results of the SPI analysis. **(A)** The AUC of the ROCs computed using the mean SPI as a function of the two parameters in the algorithm, α and Δf, plotted using a color scale with AUC = 0.5 corresponding to blue and AUC = 0.9 corresponding to yellow. **(B)** As in A but now using the median value of the SPI. **(C)** As in A but now using the proportion of channels with SPI = 1.

We computed the SPI for each patient as the mean value over all electrograms as a function of the two parameters α and Δf in the analysis (Methods). After having obtained the SPI values for both patient groups, we determined the ROC and corresponding AUC. The result of this grid search is presented in Figure 5A, where we plot the AUC using a color scheme with low/high values shown in blue/yellow. The maximum AUC value was found to be 0.85 ([0.68, 0.95] 95% CI) corresponding to a threshold of *α* = 0.18 and a frequency interval around the DF of Δf = 3.6 Hz. For these parameter values, the mean SPI for the termination patients was 0.85 (0.80–0.92) while for the non-termination patients it was 0.97 (0.93–0.98) (*p* < 0.001). This significance was retained when adding any of the patient characteristics to the SPI in a logistic regression model. After removing one outlier in each data set, the AUC only changed to 0.86 while the sensitivity analysis resulted in an identical AUC with a small IQR: AUC = 0.85 (0.83–0.87). We also performed this grid search using the median value of the SPI of all electrograms, with the results illustrated in Figure 5B. Using this median value, we found a maximum AUC of 0.82 ([0.65, 0.93] 95% CI) using a threshold *α* = 0.14 and a frequency interval of Δf = 4.0 Hz.

Finally, we asked whether the termination patients had more electrograms with SPI = 1 than non-termination patients. To address this question, we calculated for each patient the proportion of electrograms with an SPI value equal to 1, again using a grid search in threshold and frequency interval. For this comparison, we found a maximum AUC of 0.86 (([0.71, 0.95] 95% CI) using a threshold of *α* = 0.2 and a frequency interval of Δf = 10.4 Hz (Figure 5C). For these parameters, the average proportion of electrograms with SOI = 1 in termination patients was 0.91 (0.65–0.96) while this value was 1.00 (0.96–1.00) for non-termination patients (*p* < 0.001).

### Electrogram quality index (EQI)

**FIGURE 6:**
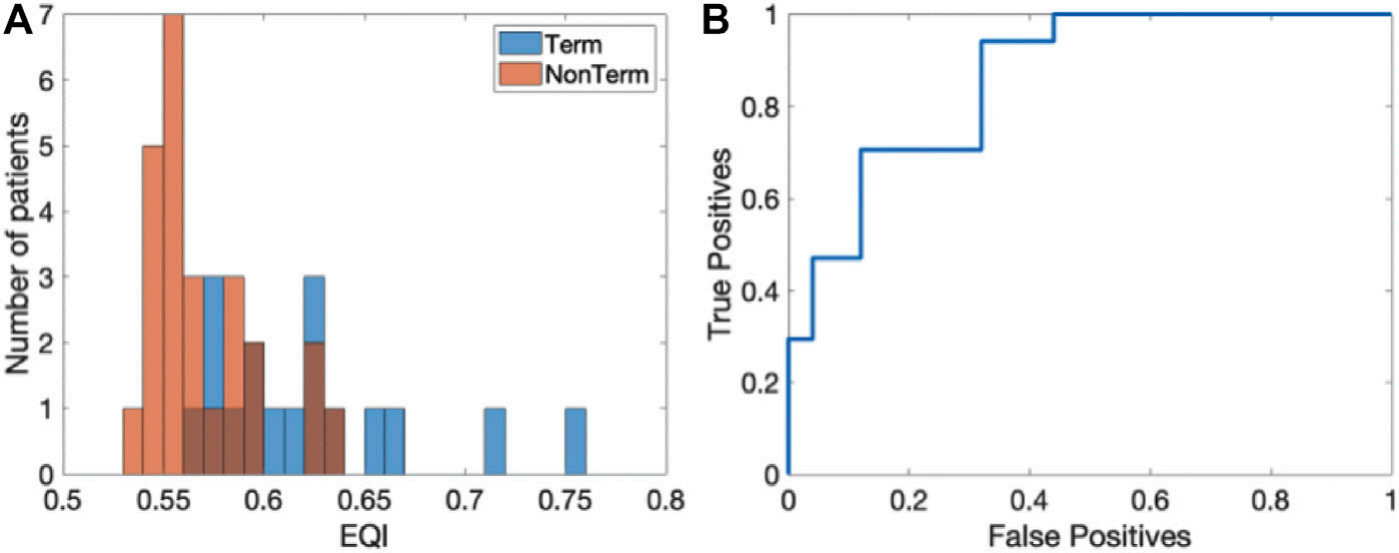
Results of the EQI analysis. **(A)** Histograms of EQI for the termination (blue) and non-termination patients (red). **(B)** ROC curve for the EQI analysis (AUC = 0.86 ([0.72,0.95] 95% CI)).

As a final quantity, we computed the EQI for each patient, averaged over all electrograms. The histograms of the EQI for the termination (blue) and non-termination patients (red) are shown in Figure 6A. The median EQI for the termination group was 0.61 (0.58–0.64) while that for the non-termination group was 0.56 (0.55–0.59) (*p* < 0.0001). As was the case for the SPI, this significance was retained when combining the EQI and any single patient characteristic in a logistic regression model. The ROC curve for this analysis is plotted in Figure 6B and has an AUC of 0.86 ([0.72, 0.95] 95% CI). Finally, one outlier was identified in the Term patients and removing this outlier did not change the AUC while the sensitive analysis resulted in an AUC value of 0.86 (0.85–0.88).

## Discussion

In this study we show that novel indices of electrograms in patients with persistent AF can identify those in whom AF did or did not terminate by ablation.

### Usefulness of AF termination

While the utility of AF termination during ablation is debated, there are few other procedural endpoints beyond isolation of the pulmonary veins. AF termination has been shown in several studies to be associated with improved long-term outcome (O'Neill et al., 2009; Scherr et al., 2015; Heist et al., 2012; Zhou et al., 2013; Park et al., 2012; Schreiber et al., 2015; Rostock et al., 2011). For example, using a stepwise ablation procedure that involved pulmonary vein isolation, electrogram-guided, and linear ablation, it was shown that inability to terminate AF during ablation was the strongest predictor of arrhythmia recurrence (Scherr et al., 2015). Other studies have failed to show this association and that further studies are required to reconcile the divergent clinical outcomes (Estner et al., 2008; Elayi et al., 2010; Wang et al., 2018). Nevertheless, termination remains the only acute endpoint that correlates with long-term outcome and knowing whether termination is achievable could enable an electrophysiologist to add or constrain lesion sets accordingly.

### Previous studies predicting AF termination

A number of previous studies have investigated whether patient characteristics can predict acute AF termination in catheter ablation. In one study, a cohort of persistent AF patients with a similar size to the current study (N = 38, with 18 termination patients) was examined (Combes et al., 2013). Several patient characteristics were found to be significantly associated with AF termination during ablation, including left ventricular ejection fraction and left atrial area. However, in a multivariable analysis, only the left atrial appendage peak flow velocity remained significant, with the termination group having a larger velocity (*p* = 0.04). The corresponding AUC was found to be 0.81. In another study, N = 70 persistent AF patients, of whom 14 terminated, were investigated (Kumagai et al., 2013). Again, it was found that the left atrial appendage contraction velocity was significantly decreased in the non-termination group and was an independent predictor of termination. No AUC value was reported but this study also showed that patients that terminated during ablation had a higher AF-free survival after 1 year than patients that needed cardioversion. In future studies, it would be interesting to combine this patient characteristic with our newly developed indices.

### Indices in our study

The ADF metric, which quantifies the frequency of the peak in the power spectrum, averaged over all electrograms, was not able to distinguish between the two patient groups. This suggests that the overall average ‘rate’ of AF within the atrium may not separate patient groups. The average DF value between the two groups (5.28 Hz vs 5.51 Hz) was not significantly different and the AUC value of the ROC was close to 0.5 (0.57). These ADF values were consistent with those reported in a recent previous study (Rodrigo et al., 2021). Note that we did not use the location of DF sites to guide ablation (Atienza et al., 2009).

Contrary to the ADF index, both the SPI and EQI had average values that differed significantly between patients with and without AF termination. These indices provided promising AUCs (0.85 and 0.86, respectively), indicating that they may be clinically useful. The SPI quantifies the amount of power contained within a certain frequency band around the DF relative to the power contained in the entire 0–20Hz interval. This SPI is a function of two parameters: the width of this frequency band and the threshold value above which the power is considered.

SPI is distinct from previous spectral organizational indices, such as those which computed a regularity index that involved the power within a fixed interval around the DF (Skanes et al., 1998; Sanders et al., 2005; Rodrigo et al., 2021). Using a systematic search, in which we varied the values of these two parameters, we found a set of parameters that optimized the AUC of the ROC. Our results indicated that, on average, the Term patients had more power in their PSD outside the interval around the DF than Non-term patients. In other words, the Non-term patients had more noisy signals, resulting in a broad peak within the interval around the DF while the Term patients had narrower peaks, with more power in harmonics that were outside this interval.

The newly introduced EQI is computed using the time derivative of the electrogram and quantifies the amplitude of the peak, relative to all other peaks in intervals of length T, the correlation time. This is equivalent to the magnitude of the slope of the electrogram, which has long been used to identify tissue activation (Colli-Franzone et al., 1982; Steinhaus, 1989; Kuklik et al., 2015). EQI is determined by first computing T in the electrogram and then, using contiguous windows of size T, determining the value of the maximum amplitude of the derivative relative to all other maxima of the derivative. Thus, an electrogram with either several near-equal valued maxima or with poorly identifiable peak values of the derivative in most intervals will have a small EQI. In contrast, very regular electrograms with a clearly identifiable and large derivative peak will result in a large EQI. We have verified that this computation is insensitive to the start time of the first time window (Supplementary Figure S1). In contrast to the SPI, the EQI does not depend on adjustable parameters and did not require fine tuning to achieve an optimal AUC. The EQI was significantly higher in the Term compared to the Non-term patients, indicating that the electrogram shapes of the Term group exhibited peaks in their time derivative that were more clearly identifiable. Activation patterns in this group determined using algorithms based on electrogram shapes may therefore be less prone to noise. This could result in better identification of rotational sources, better guidance for targeted ablation, and could result in acute termination.

Our results were computed using the simultaneous recordings from 64 electrograms, obtained from a basket catheter inserted into the left atrium. Thus, and distinct from other studies that used single recording electrodes, we were able to obtain spatially averaged quantities since this basket covers >70% of the atrium. It would be interesting, however, to apply our indices to electrograms that are collected in a pointwise fashion. Furthermore, we used recordings with a prolonged duration (60s), which reduces the likelihood of spurious results.

Our results indicate that both the SPI and the EQI may be a useful tool to predict whether ablation results in acute termination or not. Furthermore, our finding that the SPI and EQI are significantly different in Term than in Non-term patients may indicate a difference in atrial organization in these patient groups.

## Limitations

Our cohort size was moderate (N = 42) and replication in larger samples with external validation is needed. We are currently planning to expand the analysis to larger patient groups. Furthermore, we did not use the indices to prospectively predict outcome, or to guide ablation strategy. In addition, although studies have shown that acute termination during ablation correlates with long-term outcome, this study did not report any follow-up results. We are currently planning to determine whether the metrics are able to predict long-term outcome in AF patients.

## Data Availability

The raw data supporting the conclusion of this article will be made available by the authors, without undue reservation.
