# Supplementary material for: Predicting acute termination and non-termination during ablation of human atrial fibrillation using quantitative indices
Source: Front Physiol. 2022 Nov 22;13:939350. doi: 10.3389/fphys.2022.939350 (PMC9725096; doi:10.3389/fphys.2022.939350)
Supplement: Supplementary file 1 [file DataSheet1.PDF]

Supplementary Figure 1

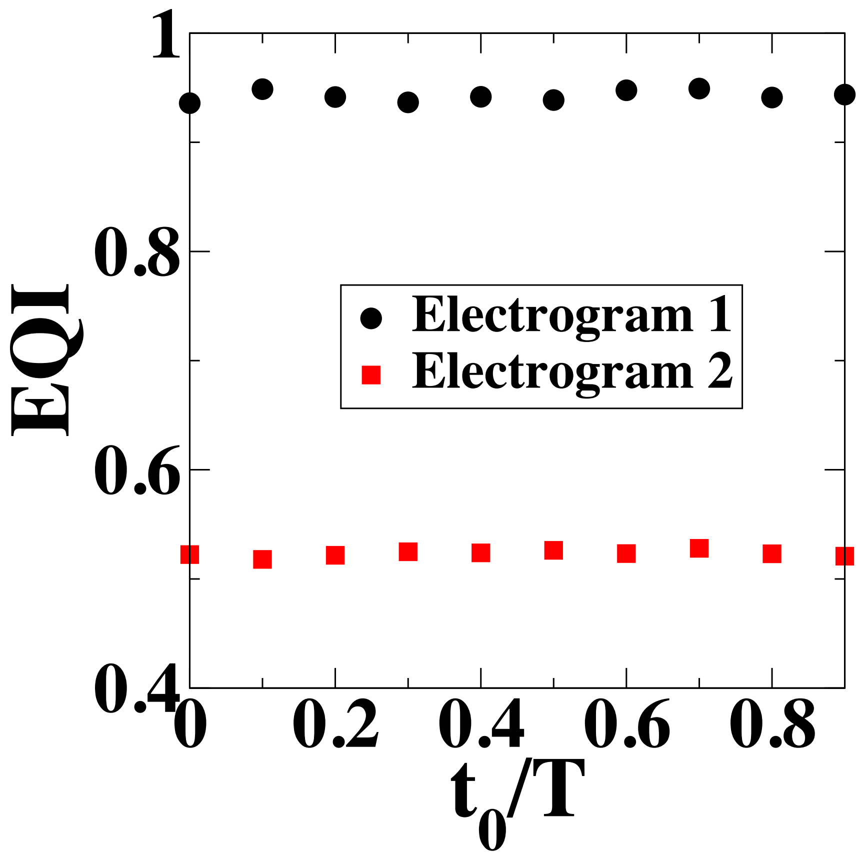

Fig. S1. The EQI for the two electrograms shown in Fig. 4 (electrogram 1 (2) corresponds to Fig. 4A (B)) as a function of the start time of the first time window t_0, normalized by the period T. The EQI did not change by more than 1.5%.
